# Characterization of the complete chloroplast genome of *Tilia taishanensis* S. B. Liang (Tiliaceae)

**DOI:** 10.1080/23802359.2020.1788430

**Published:** 2020-07-09

**Authors:** Yi-Zeng Lu, Wen-Qing Li, Ning Wang, Yan Wang, Yi Han, Tao Sun, Li-Jiang Liu, Xiao-Man Xie, Yin-Hua Wang

**Affiliations:** a Shandong Provincial Center of Forest Tree Germplasm Resources, Jinan, China; b Shandong Academy of Forest, Jinan, China

**Keywords:** *Tilia taishanensis*, chloroplast genome, phylogenetic analysis

## Abstract

The complete chloroplast genome of an endangered endemic species in China *Tilia taishanensis* was sequenced with Illumina HiSeq 2000 platform. It was a typical quadruple structure as other plants of *Tilia* with 162,803 bp in length, including a large single copy (LSC: 91,114 bp) region and a small single copy (SSC: 20,379 bp) which were separated by a pair of inverted repeats (IRa, b: 25,655 bp) region. The overall GC content is 36.5%. A total of 129 genes was annotated which contained 84 protein-coding genes, 37 tRNA genes, and 8 rRNA genes. ML Phylogenetic analysis compared with 33 expressed chloroplast genomes revealed that *T. taishanensis* was a sister to other *Tilia* species.

*Tilia taishanensis* S. B. Liang is an endangered endemic ornamental, timber and nectar plant in East China which only distributed in Shandong Province with about 500 individuals in total (Shubin 1985; Hanbin 1990; Wenqing et al. 2016; Fazeng et al. 2016). Its distribution areas are easily affected by tourism development, grazing and forest tending (Wenqing et al. 2016). And which was classified as endangered species according to the IUCN standard (Boqiang et al. 2019). The embryology (Hong 2014), cuttage breeding of *T. taishanensis* (Xiaolong et al. 2015) had been carried out. However, the understanding of its resource information was still limited. Genome mining would be helpful to the study of endangered mechanism of *T. taishanensis* and promote its conservation and utilization.

The fresh leaves of *T. taishanensis* were collected from the living individual permanently conserved in the *Tilia* gene bank (36.628°N, 117.1636°E) which was grafted from the wild tree. The specimens were preserved in National Plant Specimen Resource Center (http://www.cvh.ac.cn/, barcodes SDF1009895 and SDF1009900). Total genomic DNA was extracted by the Plant DNA extraction Kit (TIANGEN, Beijing, China) according to the requirements of the reagent company. Paired-end reads were constructed according to the Illumina library preparation protocol and sequenced on an Illumina HiSeq 2000 platform. The whole chloroplast genome of *T. taishanensis* was assembled by MITObim 1.8 (Hahn et al. 2013) and wad annotated in DOGMA (http://dogma.ccbb.utexas.edu/). Maximum-likelihood (ML) phylogenetic tree with 100 bootstrap replicates was inferred using TreeBeST 1.9.2 (Vilella et al. 2008).

The chloroplast genome of *T. taishanensis* (GenBank accession number MT591529) was also a typical quadruple structure with 162,803 bp in length that contains a large single copy (LSC: 91,114 bp) region and a small single copy (SSC: 20,379 bp), which were separated by a pair of inverted repeats (IRa, b: 25,655 bp) region. The overall GC content was 36.5%. A total of 129 genes were annotated, including 84 protein-coding genes, 37 tRNA genes, and 8 rRNA genes. Among these, 7 genes had a single intron, *ycf*3 and *clpP* had two introns. The *rps*12 had four copies, and *rrn*4.5, *rrn*5, *rrn*16, *rrn*23 had two copies respectively. The chloroplast genome of *T. Taishanensis* was similar to that of other four species of *Tilia*, but there were obvious differences. ML Phylogenetic analysis compared with 33 expressed chloroplast genomes revealed that *T. taishanensis* was a sister to other *Tilia* species (Figure 1).

**Figure 1.**
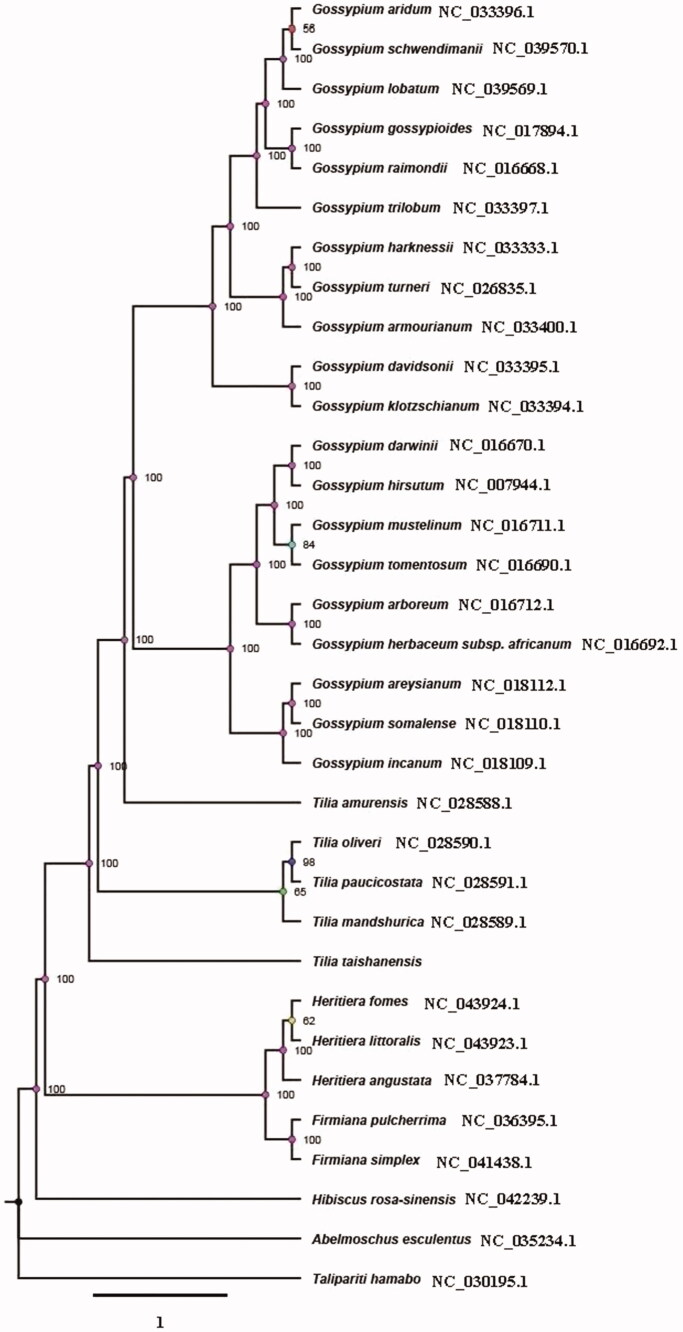
A maximum-likelihood (ML) tree of *T. taishanensis* and other 33 related species based on the complete chloroplast genome sequence. The accession numbers are showed in the figure, and the numbers behind each node are bootstrap support values.

## Data Availability

The data that support the findings of this study are available in GenBank of NCBI at https://www.ncbi.nlm.nih.gov/genbank/, GenBank accession number MT591529.
